# 

**Published:** 1845-04

**Authors:** 


CONTENTS:

Introductory, - - - 1
Case of Encephaloid Tumor of the Ovaries. By W. BUTTERFIELD, M.D., Little Fort, Ill. - - 2

BIBLIOGRAPHICAL NOTICES.
Of Puberty and the Critical Age of Women, and of the Periodical Discharge of Ova by Women and the Mammiferæ. By M. A. RACIBORSKI, M. D., &c., Paris, 1844. - - - - 4
Proof of the Periodical Ripening and Separation of the Ova of Mammiferæ and Man, independent of Coitus, being the first condition of their Propagation. By TH. L. W. BISCHOFF, M. D., P.D., Giessen, 1844, - - 8
Introductory Lectures, - - 9
An Address on Insanity, and the establishment of a Lunatic Asylum, delivered before the Committee of the House of Representatives on Education, and the public, Dec. 25, 1843, by JOHN EVANS, M. D., of Attica, Ind., - - 10
The first lines of the Theory and Practice of Surgery, including the principal operations. By SAMUEL COOPER. With notes and additions, by WILLARD PARKER, M.D., &c., - - 11

PRACTICAL MEDICINE, &c.
Miss Martineau's Marvelous Mesmerization Unmystified, - 11

MEDICAL INTELLIGENCE.
An easy and certain method of performing Catheterism, even in the most difficult cases. By J. G. MAISONNEUVE, - - 15
Blisters in Children, - - 16
Case of Singultis of several years' standing cured by Acupuncture, by Dr. EMILIANI, - - 16
New method of Dressing Wounds and Ulcers. By Dr. LAUGIER, 16

